# Working memory load reduces corticospinal suppression to former go and trained no-go cues

**DOI:** 10.1038/s41598-021-91040-6

**Published:** 2021-06-02

**Authors:** Dominic M. D. Tran, William G. Nicholson, Justin A. Harris, Irina M. Harris, Evan J. Livesey

**Affiliations:** 1 The University of Sydney, Sydney, Australia; 2 The University of Exeter, Exeter, UK

**Keywords:** Neuroscience, Psychology

## Abstract

Environmental cues associated with an action can prime the motor system, decreasing response times and activating motor regions of the brain. However, when task goals change, the same responses to *former* go-associated cues are no longer required and motor priming needs to be inhibited to avoid unwanted behavioural errors. The present study tested whether the inhibition of motor system activity to presentations of former go cues is reliant on top-down, goal-directed cognitive control processes using a working memory (WM) load manipulation. Applying transcranial magnetic stimulation over the primary motor cortex to measure motor system activity during a Go/No-go task, we found that under low WM, corticospinal excitability was suppressed to former go and trained no-go cues relative to control cues. Under high WM, the cortical suppression to former go cues was reduced, suggesting that the underlying mechanism required executive control. Unexpectedly, we found a similar result for trained no-go cues and showed in a second experiment that the corticospinal suppression and WM effects were unrelated to local inhibitory function as indexed by short-interval intracortical inhibition. Our findings reveal that the interaction between former response cues and WM is complex and we discuss possible explanations of our findings in relation to models of response inhibition.

Environmental cues that have a learned association with going or stopping can come to elicit action tendencies that speed or slow responses accordingly. Everyday examples of conditioned action tendencies include automatically breaking when seeing a stop sign at an intersection or walking on the sound of a pedestrian signal before checking the signal is green. In the laboratory, early research on *motor affordances* found that response times (RTs) were faster when the orientation of graspable objects (e.g., mugs) were congruent with the responding hand^1^, suggesting that objects can automatically prepare actions. Similarly, evidence of *action tendencies* has been observed through the modulation of RT to an imperative go cue, which decreases when a preceding warning cue is repeatedly paired with going but increases when the preceding warning cue is repeatedly paired with stopping^2,3^. As a note, we define action tendencies as an increase in the preparedness to act, move, or respond that is elicited by motor-associated cues; and we refer to motor affordances as a property of cues or objects that have had extensive (sometimes lifelong) motor associations, which likely started as an action tendency.

Neuroimaging studies have revealed that the influence of motor affordances and action tendencies on behaviour can also be observed as distinct neural signatures. For example, images of graspable objects or items supporting “utilization behaviours” (e.g., tools) have been shown to increase activity in motor regions of the brain as measured by PET^4^, fMRI^5^, and EEG^6^. Transcranial magnetic stimulation (TMS) has also been used to probe the cognitive and neural mechanisms underlying motor affordances and action tendencies^7–10^. The benefit of using TMS to study action tendencies is that it can directly measure motor system activity in a temporally precise manner. When TMS is applied over the primary motor cortex (M1), it activates peripheral muscles and this activity can be recorded with electromyography (EMG) as motor-evoked potentials (MEPs). Variations in MEP amplitudes reflect variations in corticospinal excitability and provide an index of motor preparation and inhibition^11^ at the exact time of stimulation.

Recording MEPs as a way of measuring motor system activity has been combined with a range of tasks^12–14^ to study action tendencies. Such techniques are particularly useful compared with purely behavioural experiments as they allow the quantification of subthreshold motor provocations or priming, i.e., increases in motor activity resulting from the processing of external stimuli that prime the motor system to execute an action without necessarily eliciting that action. For example, previous research has shown that MEPs probed during the presentation of a warning cue increase when that warning cue is repeatedly paired with a go cue^15^. These results demonstrate that associating a stimulus (i.e., the warning cue) with an imperative signal to respond is sufficient to reveal subthreshold motor provocations to that stimulus. Critically, participants do not respond during the warning cue, but the increase in activity reflects an increase in motor preparation for the upcoming imperative signal. Likewise, research has also shown that MEPs probed during the presentation of a warning cue decrease when that warning cue is repeatedly paired with a no-go cue^16^. This finding demonstrates that associating a stimulus with a signal to withhold responding can lead to a reduction in motor system activity elicited by that stimulus, indicating a decrease in motor preparation (or increase in motor inhibition).

Most past studies investigating the influence of warning and response cues on motor system excitability have focused on the foreperiod before response execution, while the response itself is still task-relevant and being deliberately prepared by the participant. Much less work has examined the influence of these cues when task goals change, such as when the same responses to the warning or response cues are no longer required, and the cues are thus made irrelevant. This is an important research question as we live in a dynamic environment with constantly changing goals that require us to adjust our actions appropriately, for example, when we want to retrain maladaptive behaviours. One method used in previous research to examine the effect of changing task goals on trained response cues is to implement a cue-response mapping switch. For example, past studies^17,18^ trained participants in a first phase to quickly respond to one set of images (e.g., living things = go), but to not respond to another set of images (e.g., non-living things = no-go). In a second phase the cue-response mappings were switched such that the trained go images were now no-go cues, and the trained no-go images were now go cues. The authors found that during the second phase, RTs to former no-go (now go) cues were slowed and commission errors to former go (now no-go) cues were increased relative to cues that did not have their mappings switched.

Similar studies have also been conducted that directly investigate the role of the motor system when task goals change. We have documented that former go (now no-go) cues trained within a 1 h session show increased motor excitability relative to control cues^19^. That is, stimuli that were previously imperative cues can elicit action tendencies even though they are no longer response cues under a new set of task rules. However, a critical feature of this finding was that the subthreshold motor provocations were largely dependent on the new task rules. When participants were asked to count the former go cues (and were not required to make any form of online response), we observed evidence for residual motor preparation—MEPs increased during presentations of the former go cues. However, when participants were required to respond to a new set of go cues while ignoring the former go cues, we observed evidence of motor inhibition—MEPs decreased during presentations of the former go cues. We speculated that this inhibition was evidence for top-down, wilful, and goal-directed cognitive control (likely originating from the frontal cortex^20–22^) and was implemented to minimise unwanted behavioural errors to former response cues while engaging in a new response task. Consistent with this postulation, the corticospinal suppression was absent during a passive counting task where there was no need to withhold responding or any possibility of making commission errors, which allowed the conditioned action tendencies to be revealed. These results suggest that simply encountering former response cues can activate the motor system and consequently make the task of retraining behaviours more challenging, but that one way of gaining behavioural control is for top-down processes to inhibit motor activity and ensure excitability remains below the threshold for actual movements.

In the present study, we further explored the cognitive and neural mechanisms underlying the modulation of motor system excitability by former go cues. Specifically, we focused on the corticospinal suppression elicited by former go cues when participants were engaged in a response task with a new set of go cues. We hypothesised that if this suppression is modulated by top-down cognitive control processes then it should be reduced with a working memory (WM) load. There is a multitude of research on the use of WM manipulations to tax executive function^23,24^. For example, individual differences in WM capacity or impairments due to aging are associated with deficits in using a top-down goal-directed response strategy and increased reliance on a bottom-up cue-elicited response strategy^25,26^. Likewise, experimental manipulations that vary access to executive function find that imposing a WM load, such as through the addition of distractor items, lead to an increase in the number of response errors that require cognitive control to inhibit^27^. Notably, these studies show that when WM is impaired, responding is not random but typically follows a cue-based strategy reflective of statistical probability and training history. In the same manner, the present study tested whether a WM load would release the corticospinal suppression elicited by former go cues and reveal an action tendency (i.e., increase motor excitability) that was otherwise being actively inhibited during a new response task.

To our knowledge, only one study has previously examined the effect of WM load on action tendencies with neuroimaging techniques. Using EEG markers of motor preparation, previous research found that a high WM load reduced the motor response to action-affording images that were irrelevant to the participants’ primary task^28^. That is, contrary to the authors’ own predictions and the direction of our current hypothesis, they found that WM load reduced, rather than enhanced, the motor affordance effect of irrelevant motor provoking stimuli. The authors showed in a follow-up experiment that operating under high WM load produced sustained inhibitory activity in the motor system as indexed by a paired-pulse TMS measure, long-interval cortical inhibition (LICI), which is thought to be mediated by GABA_B_ neurotransmission in M1^29,30^. As a result, the authors posited that an inhibitory state was induced under a high WM load to prevent the affordance effect from activating the usual action tendencies in the motor system. However, the authors based this conclusion on two separate experimental results and did not retest the WM affordance affect when testing the impact of WM on LICI^28^. Given the unexpected results in past research^28^, our previous findings^19^ provided a useful paradigm for examining the effects of WM on action tendencies in a more controlled manner by probing former response cues trained in the laboratory.

In addition to examining the impact of WM on the corticospinal suppression elicited by former go cues, we also aimed to investigate the mechanisms underlying this suppression effect. Previous research^28^ showed that LICI was a useful candidate for inspecting the inhibitory state of the motor system under a high WM load. However, the inhibitory state elicited by task-irrelevant cues observed in our past findings^19^ is much more selective, which contrasts with the more “sustained” inhibition observed in past research^28^. Given this difference, we reasoned that short-interval intracortical inhibition (SICI) would be a better candidate for inspecting the transient inhibitory state of the motor system elicited by former go cues. SICI is a paired-pulse TMS measure that indexes a reduction in the amplitude of MEPs elicited by a suprathreshold TMS pulse when it is preceded by a subthreshold conditioning pulse 1-6 ms earlier. The magnitude of the MEP suppression by the preceding conditioning pulse is thought to be mediated by GABA_A_ neurotransmission in M1^31^. Past research has demonstrated that SICI is reliable^32^ and sensitive to transient inhibitory states and fluctuations in motor excitability elicited by imperative cues. In the stop signal task, SICI increases prior to stopping during presentations of a stop cue and decreases prior to going during presentations of a go cue^33^. These findings reveal that stopping and going in response to imperative cues involve the recruitment and disengagement, respectively, of local inhibitory circuits in M1 as indexed by SICI. Therefore, we measured SICI to track the possible recruitment and disengagement in inhibitory states to former go cues under low versus high WM load.

The research aims were investigated in two experiments. Experiment 1 manipulated WM load to examine the impact of cognitive control on motor system excitability elicited by former go (now no-go) and trained (consistent) no-go cues. Based on our past findings^19^, we predict that under low WM load, former go and trained no-go cues will show reduced motor excitability compared to control cues. Critically, we hypothesised that the reduction in motor excitability to former go cues is driven by top-down cognitive control processes, which will be impaired under high WM load. Hence, we predict that under high WM load, the motor suppression to former go cues will be reduced, while the motor suppression to trained no-go cues, which is less reliant on cognitive control, will be less affected.

Experiment 2 manipulated WM load in the same manner as Experiment 1, but additionally measured SICI to examine possible mechanisms underlying the corticospinal suppression elicited by former go and trained no-go cues. We predict that under low WM load, there will be more SICI to former go than control cues. Under high WM load, we predict that the amount of SICI to former go cues will be reduced compared to levels under low WM load. That is, for former go cues, we hypothesise that SICI will follow the reverse pattern to motor excitability: under low WM load, when excitability is low (suppression), we expect more SICI; and under high WM load, when excitability is higher (reduced suppression), we expect less SICI. For trained no-go cues, we describe two alternative hypotheses: one possibility is that SICI levels under low versus high WM load will mimic the pattern predicted for former go cues (i.e., more SICI under low WM than high WM). Another possibility is that well-trained (no-go) cues are less reliant on the recruitment of local inhibitory circuits in M1 to reduce motor excitability, and thus there will be low levels of SICI regardless of WM load.

## Results

**Figure 1:**
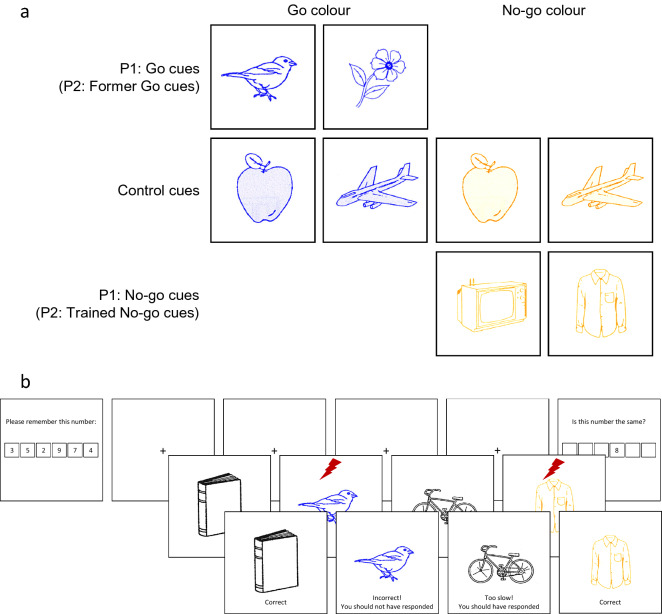
(**a**) Example design of Experiments 1 and 2 Phase 1 (P1) with blue as the go colour and yellow as the no-go colour. Assignment of go/no-go colours and allocation of the six selected images to the six critical cues were randomised between participants. (**b**) Schematic of Experiments 1 and 2 Phase 2 (P2) with the working memory task interleaved between four go/no-go trials. Black images were assigned as the go colour; blue and yellow images were assigned as the no-go colour. TMS (red bolt) was triggered 300 ms after the onset of each critical cue. In Experiment 1, all TMS trials were single pulse (suprathreshold) TMS. In Experiment 2, half the TMS trials were single pulse (suprathreshold); the other half were paired pulse with the suprathreshold pulse preceded by a subthreshold pulse 2.5 ms earlier to measure SICI.

Experiments 1 and 2 examined the impact of WM load on motor system excitability to imperative cues in a Go/No-Go task following a rule switch. Participants were first trained (Phase 1) on a set of six cues using a Go/No-Go task with blue and yellow images. During this first phase, two images were always presented in the assigned go colour (go cues), two images were always presented in the assigned no-go colour (no-go cues), and two images appeared in both colours an equal number of times (control cues; Fig. 1a). In Phase 2, participants completed a Go/No-Go task with new rules in addition to a WM task, and received TMS. For the new Go/No-Go task, participants were instructed that black (a novel colour) was now the go colour and that both blue and yellow were now no-go colours. The WM task was manipulated within-participants and blocks alternated between low and high WM load trials (see Methods section for further details). Single-pulse TMS was triggered during the presentations of the three critical cue conditions, which were now all no-go cues under the new task rules: former go cues, trained no-go cues, and control cues (Fig. 1b).

### Effect of rule switching and working memory on error rates and response times

**Figure 2:**
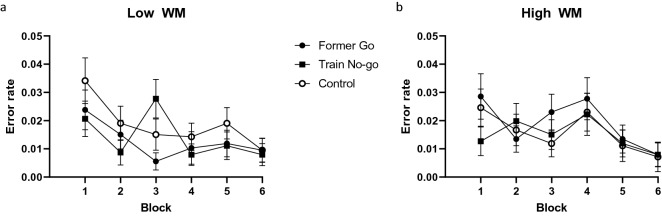
Commission errors during Phase 2 pooled from Experiments 1 and 2. Error rates for cue type by block separated for low (**a**) and high (**b**) working memory load. Error bars represent within-participant standard errors. Note that Experiment 2 Phase 2 was longer than Experiment 1 Phase 2 due to the addition of paired pulse TMS trials. The Phase 2 data were split into 6 equal blocks in each experiment for graphical representation and statistical analysis.

Commission (false alarm) errors for the three critical cue conditions during Phase 2 (after the rule switch) are presented in Fig. 2. Error rates for these no-go cues were low overall and decreased across blocks but the floor effect made it difficult to detect differences between cue types. There was a significant effect of block (F(5,520) = 5.62, *p* < 0.001, η^2^_p_ = 0.05), and no significant effect of cue type, WM, two-way cue type × block interaction, or three-way cue type × block × WM interaction (F’s < 1). The two way WM interactions with cue type (F(2,208) = 2.16, *p* = 0.118, η^2^_p_ = 0.02) and block (F(5,520) = 2.02, *p* = 0.075, η^2^_p_ = 0.02) were also non-significant. As expected, errors occurred most frequently immediately after the switch during block 1 of Phase 2. A contrast comparing cue types that were previously (always or sometimes) go cues during Phase 1 (former go and control) against cues types that were previously no-go cues during Phase 1 (trained no-go) revealed no significant difference under low WM (t(104) = 1.09, *p* = 0.276, d = 0.11) and a trend under high WM (t(104) = 1.86, *p* = 0.065, d = 0.18). Phase 2 RTs for the new go cues by WM condition were M_low_ = 579.55 ms, and M_high_ = 584.50 ms (SEM_within_ = 1.30). There was a very small but significant effect of WM load in slowing RT (F(1,104) = 7.27, *p* = 0.008, η^2^_p_ = 0.07). The behavioural results show that switching the cue-response mappings in the task did not affect error rates. Although there was some indication that high WM might differentially increase errors for cues that were previously associated with the go response, none of the relevant statistics were significant and on the whole error rates were close to floor. This highlights a key advantage of using TMS to measure corticospinal excitability. Doing so allows us to measure action tendencies even in the absence of overt behavioural errors.

### Effect of working memory on motor excitability to former go and trained no-go cues

**Figure 3:**
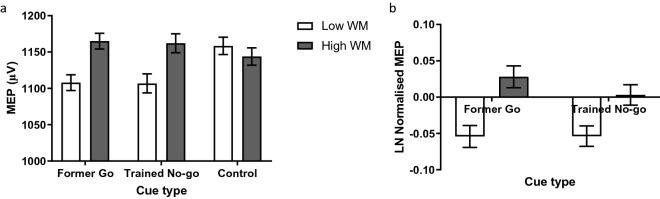
(**a**) Mean raw MEP of data pooled from Experiments 1 and 2 by working memory and cue type. Simple effect of working memory on control cue: t(104) = 0.62, *p* = 0.539, d = 0.06, BF_01_ = 7.68. (**b**) Mean MEP log-normalised to the control cue of data pooled from Experiments 1 and 2 by working memory and cue type. Error bars represent within participant standard errors across working memory conditions separately for each cue type.

Raw single pulse MEPs pooled for Experiments 1 and 2 are presented in Fig. 3a. Mean MEPs for former go and trained no-go cues were normalised to the mean MEP of control cues for each participant; this value was then log transformed (see Methods). As predicted, under a low WM load, motor system activity elicited by former go and trained no-go cues was suppressed relative to control cues. Also as predicted, under a high WM load, the suppression to former go cues was absent, with motor system activity to former go cues elevated relative to control cues (Fig. 3b). Unexpectedly, however, the WM manipulation also had the effect of reducing the corticospinal suppression to trained no-go cues. We found a significant main effect of WM (F(1,104) = 7.08, *p* = 0.009, η^2^_p_ = 0.06), and no significant effect of cue type (F(1,104) = 0.62, *p* = 0.431, η^2^_p_ = 0.01) or WM × cue type interaction (F(1,104) = 0.93, *p* = 0.337, η^2^_p_ = 0.01). There were also significant simple effects of WM on former go cues (t(104) = 2.72, *p* = 0.008, d = 0.27) and trained no-go cues (t(104) = 2.01, *p* = 0.047, d = 0.20). The difference in motor system activity between former go cues under low versus high WM is consistent with our hypothesis that the corticospinal suppression to former go cues would be reduced with a WM load. However, we also observed a similar, albeit (numerically) weaker, effect for trained no-go cues (and failed to find a significant WM × cue type interaction), suggesting that the effect of WM load in reducing corticospinal suppression was not selective to former go cues.

One sampled t-tests were conducted on the log-normalised MEP comparing cue type at each WM level to a value of zero (Table 1). Corticospinal suppression to former go and trained no-go cues under low WM were both significantly below baseline, while motor excitability to former go and trained no-go cues under high WM was not significantly different from baseline. In summary, motor system activity to former go and trained no go cues was suppressed during a response task, and WM load had a general effect of reducing the motor suppression to both these cues.

**Table 1:** One sample t-tests (H_0_ = 0) of log-normalised MEP (Fig. 3b). Data were pooled from Experiments 1 and 2 for cue type × working memory load.

WM	Cue type	t	*p*	d
Low	Former Go	−2.183	0.031*	−0.213
	Trained No-go	−2.523	0.013*	−0.246
High	Former Go	1.475	0.143	0.144
	Trained No-go	0.162	0.871	0.016

*p < 0.05.

### Effect of working memory on SICI to former go and trained no-go cues

**Figure 4:**
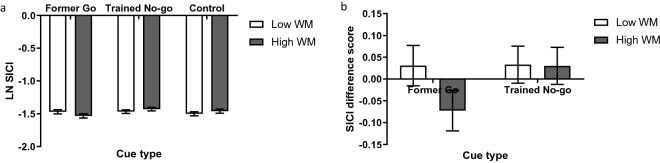
(**a**) SICI represented as mean log(paired-pulse MEP/single-pulse MEP) by working memory and cue type. (**b**) Mean SICI difference scores from the control cue by working memory and cue type. Error bars represent within participant standard errors across working memory conditions separately for each cue type.

In Experiment 2, we additionally measured SICI to investigate if the corticospinal suppression to former go and trained no-go cues was mediated by local inhibitory circuits in M1. SICI was calculated as the log ratio of mean paired-pulse MEP over the mean single-pulse MEP for each participant (Fig. 4a), with more negative values indicating that there was more SICI and therefore more recruitment of local motor inhibition. We found significant SICI (i.e., a reduction of paired-pulse MEPs relative to single-pulse MEPs) across all WM and cue type conditions (Table 2). Unexpectedly, there was no significant effect of WM or cue type on SICI; even the control cues induced a similar level of SICI compared to the former go and trained no-go cues. Analysing the mean SICI difference score from the control cue (Fig. 4b), there was no significant effect of WM (F(1,50) = 0.48, *p* = 0.493, η^2^_p_ = 0.01; BF_01_ = 4.61), cue type (F(1,50) = 1.04, *p* = 0.313, η^2^_p_ = 0.02; BF_01_ = 4.23), or WM × cue type interaction (F(1,50) = 1.24, *p* = 0.271, η^2^_p_ = 0.02; BF_01_ = 3.21). The pattern of results and statistical outcomes were similar if we analysed mean SICI log-normalised to the control cue, matching the analysis performed on single pulse MEPs, however this analysis involves two steps of log-normalisation and can be complex to interpret. In summary, SICI was observed for all critical cues during Phase 2 of the Go/No-go task. WM and cue type did not have a differential effect on SICI, with substantial evidence supporting the null hypothesis (compared to the alternative hypothesis).

**Table 2:** One sample t-tests (H_0_ = 0) of log SICI data (Fig. 4a) for cue type × working memory load.

WM	Cue type	t	*p*	d
Low	Former Go	−17.440	< .001***	−2.488
	Trained No-go	−16.113	< .001***	−2.442
	Control	−17.768	< .001***	−2.256
High	Former Go	−16.398	< .001***	−2.488
	Trained No-go	−16.313	< .001***	−2.296
	Control	−17.661	< .001***	−2.284

SICI = ln(single-pulse MEP/paired-pulse MEP).

***p < 0.001.

## Discussion

The present study examined the effect of WM load on motor system activity in response to presentations of former go and trained no-go cues. We showed that under low WM load, former go cues and trained no-go cues produced significant suppression of motor activity relative to the effect of control cues. These results are consistent with previous reports^19^ that former go cues require corticospinal suppression during a response task with a new set of rules or cue-response mappings. Further, we found that under high WM load, the corticospinal suppression to both former go and trained no-go cues was significantly reduced. Notably, the corticospinal suppression was not a general effect of WM load on motor system excitability, as reflected by the lack of difference in raw MEP amplitudes on the control cues across WM condition; rather, the suppression was a specific effect on former go and trained no-go cues. The reduction in suppression from low to high WM was numerically greater for former go cues than trained no-go cues, but this difference (i.e., the interaction) was not statistically significant. A second question of interest was whether the loss of corticospinal suppression would reveal action tendencies to the former go cue that were inhibited under low load. Motor system activity to former go cues was elevated above baseline under high WM load, however, this level of excitation was not statistically significant. While we found no effect of cue type or WM on error rates during Phase 2, there was a marginal effect of high WM load on cue type during the first block following the rule switch, and the MEP results indicate that cue type and WM modulated motor preparation even in the absence of overt errors. Finally, in Experiment 2 we also examined whether the reduced motor activity elicited by former go and trained no-go cues involved changes to SICI and how this form of inhibition was affected by WM load. We found significant levels of SICI across all conditions but failed to find any evidence that SICI was modulated by either WM load or by cue type.

Under a low WM load, decreased motor system excitability was observed to former go and trained no-go cues relative to control cues that were all trained in Phase 1 and assigned to be no-go cues in Phase 2. Note that the suppression to the former go and trained no-go cues was not simply a product of the no-go assignment of these cues in Phase 2, because this was also true for the control cues. The control cues were matched for frequency of presentation in Phase 1, they merely differed in that they were not uniquely predictive of either going or stopping, having been presented in both colours during Phase 1. One explanation for observing reduced motor excitability to former go cues is that it reflects a top-down cognitive control process intended to suppress any action tendencies elicited by cues that previously signalled the need for an imperative go response. This inhibitory command likely originates from the frontal cortex^20–22^ and recruits the inferior frontal gyrus^34–37^ and supplementary motor area^38,39^. Consistent with this view, we found that a WM load manipulation, which is often used to deplete executive functioning resources, reduced the corticospinal suppression to former go cues. Under high WM load, motor excitability was numerically elevated for the former go cue relative to baseline, but this difference was not statistically significant. It should be noted that elevation of motor excitability above baseline in these types of designs that probe motor activity to former imperative cues^19^ is difficult to detect statistically as the effect sizes are generally small. Even though we had a large sample size (100 + participants), more than most TMS studies, considering that the former go cues are novel images trained within a 1 h Go/No-Go task, it is not surprising that any subthreshold motor provocations would be small. We expect that environmental cues with a lifetime of training or extended cue-response pairings would elicit much greater excitation levels under high WM load and therefore be more difficult to withhold responding or maintain goal-directed behaviour.

The changes in motor system excitability to trained no-go cues are somewhat more complex to interpret. We had expected that the suppression underlying trained no-go cues would be less reliant on top-down control, as it would be driven in part by bottom-up or automatic inhibition elicited by the cues due to their no-go training history from Phase 1. Hence, our prediction was that this automatic inhibition would be less impacted by the WM load manipulation than the suppression to former go cues. To some extent, this hypothesis was supported by the pattern of results, although the difference (i.e., the interaction) was not statistically significant. There are two possible interpretations of the finding. First, the corticospinal suppression to trained no-go cues may comprise both bottom-up and top-down inhibition—partly driven by the cue as a consequence of training and partly driven by the participant’s goal-directed strategy to withhold responding. Examples of such a strategy could involve adopting a task rule or motor command to “continue not responding to the no-go cues” or “withhold responding to any familiar image-colour combinations”. Second, the suppression to trained no-go cues could be entirely driven by top-down processes, and hence is not distinguishable from the processes underlying suppression to former go cues. However, we think this latter account is unlikely, given previous findings of automatic behavioural^17^ and cortical^40^ inhibition. Therefore, it is likely that the mechanisms controlling the suppression of well-trained inhibitory responses also, partly but not entirely, rely on executive processes that are taxed by WM.

Our paired-pulse TMS findings revealed robust SICI in the presence of former go, trained no-go, and control cues. This aspect of the results was largely expected given that the critical cues were all no-go cues in Phase 2 and there is extensive evidence linking SICI and response inhibition^32,33^. However, SICI was not additionally modulated by cue type or WM load. One possible explanation for this outcome is that the amount of SICI during no-go trials overwhelmed any difference that may be elicited due to the (Phase 1) training history of the cues or the WM manipulation. Alternatively, the lack of SICI effects could suggest that fluctuations in the MEPs, including suppression of former go and trained no-go cues under low WM, is independent of GABA_A_ function within motor cortex. Our results show that single-pulse MEP measurements are more sensitive to cue-response training and WM load manipulations than SICI measurements. Single-pulse MEPs index the overall activity of the corticospinal pathway^41^ while SICI indexes the amount of local inhibitory tone in M1 mediated by GABA_A_ activity. Therefore, our findings suggest that during a response task requiring cognitive control, WM load reduces overall corticospinal suppression throughout the motor system (from spinal motoneurons through to upstream networks connected to M1), rather than specifically disengaging local inhibition in M1.

The WM results are consistent with our hypothesis that top-down control processes mediate motor system activity to former go cues. However, on face value, the results are inconsistent with past research showing that WM load increased inhibition, rather than released inhibition^28^. This past study showed that EEG markers of the motor affordance effect were reduced by a WM load, and separately that WM load increased inhibitory tone in M1 as measured by LICI. Notably, these results were also contrary to the authors’ own predictions about how WM would impact the motor affordances. The authors had hypothesised an effect of WM load more akin to what we found—specifically, increased affordance-related motor excitability under high WM load compared to low WM load. One difference in our designs that may account for the different findings is the task participants were engaged in while motor preparation was probed. In the previous experiments^28^, the authors used a passive WM task in which irrelevant images of affording objects were presented between the study and test trials; we used a Go/No-go task in which go cues during training were reassigned as no-go cues at test. However, research has shown that the current task context can have large influences on the neurophysiological indices of motor preparation. For instance, former go cues induced elevated motor excitability (i.e., action tendency) if participants were counting but not responding, while former go cues induced suppressed motor excitability (i.e., response control) if participants were responding to novel go cues^19^. In addition to this difference in the tasks used (to present the motor provoking stimulus) is the starting point of the WM control condition. Past research showed a motor affordance effect under low WM load and found this to be reduced under high load^28^, whereas we showed motor excitability was suppressed under low load and found this to reveal a motor affordance-like effect under high load. Therefore, whether or not action tendencies are activated or inhibited under low WM load, may also be critical in determining the current cognitive processes engaged and how high WM load will ultimately impact these processes.

The key finding from the present study is that corticospinal suppression to former go cues and trained no go cues was reduced by a WM load. Although the precise neurocircuitry contributing to the suppression and the release of suppression are still to be determined, they likely involve recruitment of the frontal cortex, are disrupted under high WM load, and bypass circuits responsible for SICI. These results also have implications for the management of motor control in the real world. They are particularly relevant in situations that require goal-directed response inhibition over habitual actions, such as driving on the opposite side of the road while travelling in a new city; or suppressing the urge to eat junk food while trying to adhere to a healthier diet. Our results indicate that the neural mechanisms that underpin our ability to apply restraint over the incorrect or unwanted action tendencies in such situations are less effective in settings that are highly distracting (e.g., when having a conversation while driving, or when watching television and snacking).

## Materials and methods

### Participants

#### Experiment 1

Students from The University of Sydney participated in exchange for course credit or remuneration. A safety screening questionnaire was signed, and informed consent was obtained from all participants before starting the experiment. All procedures were approved by The University of Sydney Human Research Ethics Committee and all methods were carried out in accordance with these guidelines and regulations. We aimed to collect data from 60 participants. Two participants were tested on a pilot version of the experiment; we have full data from 58 participants. Of the 58 participants, one was excluded for having a low number of recorded MEPs (less than half of the total MEP trials were > 50 µV), one was excluded based on WM performance (< 60% accuracy), and four were excluded for having log-normalised MEPs greater than 3 standard deviations from the mean of all participants (see Data Analyses and Statistics section for normalisation method).

#### Experiment 2

Recruitment procedures were the same as for Experiment 1. We aimed to collect data from 60 participants but had to stop after 58 participants, as face-to-face laboratory testing was suspended due to COVID-19. Of the 58 participants, two withdrew before completing the experiment (one was uncomfortable with the sensations produced by the TMS pulse over the scalp, and one needed to leave early), one was excluded based on test performance (> 3 standard deviations from the mean for commission and omission errors), and two were excluded for log-normalised MEPs greater than 3 standard deviations from the mean of all participants.

### Experiment setup

The experiment setup was similar to that used in our previous research^19^. TMS was administered using a Magstim 200^2^ (Experiment 1) or BiStim^2^ (Experiment 2) with a 70-mm figure-eight coil (Magstim, Whitland, UK). Pulses were delivered to left M1 with the coil positioned in a posterior–anterior configuration and the handle oriented approximately 45° from the midline. In preparation for electrode placement onto the hand, the skin was cleaned with a small sponge and wiped with 70% v/v isopropyl alcohol swab. Surface EMG traces were recorded from the first dorsal interosseous (FDI) muscle of the right hand, using a pair of Ag/AgCl electrodes placed in a belly–tendon arrangement over the muscle and a ground electrode placed over the ulnar styloid process of the wrist. Data from 200 ms pre-stimulation to 100 ms post-stimulation were collected via a PowerLab 26 T DAQ device (ADInstruments, Bella Vista, NSW, Australia). The analogue EMG signal was digitized (sampling rate: 4 kHz; bandpass filter: 0.5 Hz to 2 kHz; mains filter: 50 Hz) and stored on a computer using LabChart software (Version 8, ADInstruments) for offline analysis. Timing between TMS and EMG was synchronized through TTL signals sent from the test computer to the Magstim unit and PowerLab.

The location of the motor cortex “hotspot” was determined starting from a position 5 cm lateral and 1 cm anterior to Cz. The experimenter moved the coil around the starting position until the maximal MEP was elicited in the FDI muscle. A fitted cap marked with the 10–20 EEG locations was worn by participants to aid in this process. Once the hotspot was located, an adjustable forehead and chin rest was used to minimise any head movements and the coil was locked in position using a mechanical arm (Manfrotto, Cassola, Italy).

Resting motor threshold (rMT) was defined as the lowest stimulation intensity capable of inducing MEPs with a minimum of 50 μV peak-to-peak amplitude in 5 of 10 consecutive pulses^42^. Single-pulse intensity during Experiments 1 and 2 was set to 120% of rMT. Paired-pulse intensity during Experiment 2 was set to 80% of rMT for the conditioning pulse (S1) and 120% of rMT for the test pulse (S2). The interstimulus interval between S1 and S2 was 2.5 ms. For Experiment 1, the mean rMT was 47.00% (SD = 8.54%) of the maximum stimulator output; for Experiment 2, the mean rMT was 52.13% (SD = 10.86%) of the maximum stimulator output.

The experiment was run on a Windows 7 PC using MATLAB software to control stimulus presentation and TMS delivery. Stimuli were close to 300 × 300 pixels in size and were displayed at the centre of the screen on a 24-inch ASUS monitor (1920 × 1080 resolution, 60 Hz refresh rate) from a viewing distance of ~ 57 cm.

### Experiment procedure

The testing sessions for each of the experiments were approximately 2 h long. The session comprised of briefing and setup (15 min), Phase 1 behavioural training (60 min), Phase 2 TMS testing (Experiment 1: 30 min, Experiment 2: 45 min), and debriefing (5 min).

#### Go/no-go task and TMS design

The reaction time task required participants to press the spacebar or to withhold a response depending on the colour of a presented stimulus. In Phase 1, images were either displayed in blue or yellow, with one colour requiring a speeded response (“go” colour) and the other requiring no response (“no-go” colour). Colour allocation was randomised between participants. The task contained a subset of 20 images selected from a standardised set of pictures^43^. Six images were designated critical cues that were assigned to be one of the cue type conditions (2 × go cue, 2 × no-go cue, 2 × control cue). During Phase 1, go cues always appeared in the go colour, no-go cues always appeared in the no-go colour, and control cues appeared in the go and no-go colours each half the time. The allocation of the six images to cue type was randomised between participants. The remaining 14 images were filler images that randomly appeared in either colour. Control cues also differed from filler images in that they were matched to the go and no-go cues in frequency of presentation for the purposes of TMS testing in Phase 2.

Participants were instructed to respond to any picture in the go colour and not respond to any of the pictures in the no-go colour. Each trial started with a fixation cross that appeared for 250–500 ms on a white background. A coloured image was then presented for 1000 ms and participants were required to respond (by pressing the space bar) or withhold a response. A feedback screen followed for 1500 ms where the image and text feedback were displayed together. For correct (go or no-go) trials the feedback displayed was ‘Correct’. For commission errors, the feedback displayed was ‘Incorrect! You should not have responded’. For omission errors, the feedback displayed was ‘Too slow! You should have responded’. Trials were separated by a 1000 ms intertrial interval (ITI). Phase 1 contained 40 blocks of 16 trials. Each block contained two presentations of each of the six critical cues and four filler images. The phase was separated by a self-paced break after every 10 blocks, but otherwise trials were presented continuously.

In Phase 2, images were displayed in black, blue, or yellow. Black was a novel picture colour requiring a speeded response, and the other colours (blue and yellow) required no response. The six critical cues were now only presented on no-go trials in Phase 2 but differed in their training history: 2 × *former* go cues, 2 × *trained* no-go cues, and 2 × control cues. In Experiment 1, one former go and one trained no-go cue was presented in blue and the other in yellow; in Experiment 2, former go and trained no-go cues were presented in the same colour that they appeared in during Phase 1. In both experiments, one control cue was presented in blue and the other in yellow. Colour did not have a significant effect on motor excitability (see Supplementary Materials for more details). Each block in Phase 2 contained 16 trials, one presentation of each cue and 10 filler images. The reduced number of cue presentations in each block during Phase 2 compared to Phase 1 allowed time between trials for the TMS machine to recharge. TMS was triggered 300 ms after the onset of each critical cue presentation. In Experiment 1, there were 24 TMS trials for each of the six critical cues; therefore, there were 48 for each of the three cue types, and 144 TMS trials in total. All critical cue trials triggered single-pulse TMS and were equally split by WM condition (low, high). The phase contained 24 blocks separated by a self-paced break after every 8 blocks, but otherwise trials were presented continuously.

In Experiment 2, there were 36 TMS trials for each of the six critical cues; therefore, there were 72 for each of the three cue types and 216 TMS trials in total. TMS trials were equally split by TMS measure (single-pulse, paired pulse) and WM condition (low, high). TMS trials were pseudorandomised between single- and paired-pulse such that there were no more than 2 TMS trials of the same type in a row for each cue, and an equal number of paired and single pulse trials for each cue within a set of 12 trials. The single-pulse and S2 of the paired pulse occurred 300 ms after the onset of the stimulus and S1 occurred 2.5 ms before S2. The phase contained 36 blocks separated by a self-paced break after every 6 blocks, but otherwise trials were presented continuously.

#### Working memory task

The WM task started in the penultimate block of Phase 1 for participants to become acquainted with the procedure and continued throughout Phase 2. At the start of Phase 1 Block 39, participants were instructed that they would perform a secondary memory task while continuing the Go/No-go task. Before a go/no-go trial, participants were presented with either a one-digit (low WM) or a six-digit (high WM) number string for 3000 ms and were instructed to remember the number as best they could. After four go/no-go trials, participants were presented with a single digit on the screen and asked if the digit was the same as the one previously presented. The high load memory probe presented a six-digit number with five missing digits (e.g., _ 2 _ _ _ _); participants were required to answer if the digit was previously presented and in the correct position. Low and high WM blocks alternated every 16 go/no-go trials with the starting WM condition randomised between participants.

## Data analyses and statistics

Pre-processing of MEP data was conducted using custom software written in Python (https://github.com/nicolasmcnair/MEPAnalysis). TMS trials with background EMG larger than 50 μV peak-to-peak amplitude prior to the pulse were excluded. MEP amplitudes from the remaining trials were selected as the peak-to-peak difference value. For the analysis, any single pulse MEP with an amplitude less than 50 µV was considered a misfire that occurred off the hotspot location (due to participant head movement or coil movement) and was excluded from the analysis.

The single-pulse MEP data were log-normalised to the mean control cue MEP at a participant level to remove large individual differences in the absolute MEP values. We have adopted log-normalisations in our previous work^19,44^, because log-transforming the normalised values centers the mid-point around 0 and removes an inherent positive skew. In Experiment 2, paired-pulse MEP data were used to measure SICI, which was calculated as the mean paired-pulse MEP divided by the mean single-pulse MEP before taking the log of this ratio.

To increase our statistical power for detecting small-moderate MEP effect sizes and to minimise the impact of potentially high variance due to having a low number of MEP trials per cue type (when split by WM condition, and TMS measure in Experiment 2), we analysed the single-pulse data pooling Experiments 1 and 2 (n = 52, n = 53). The Go/No-go task, WM task, and single-pulse TMS parameters from Experiments 1 and 2 were almost identical. Single-pulse data were first analysed as a three factor ANOVA with Experiment (1, 2), WM (low, high), and Cue Type (Former go, Train no-go). There was no significant effect of Experiment or significant interactions with Experiment (all F’s < 1) so we subsequently removed this factor from the model. The single-pulse analyses reported in the Results section are from a two-factor model with WM (low, high) and Cue Type (Former go, Train no-go). Since paired-pulse data was only collected in Experiment 2, the data were analysed using the same two-factor model with WM and Cue Type. Bayesian statistics were included for analyses that were theoretically interesting to assess the strength of evidence for the null hypothesis. The Bayes factors reported were based on the analysis of effect across matched models and default prior set in JASP 0.12.2.

## Supplementary Information

Supplementary Information.
