# Supplementary material for: Working memory load reduces corticospinal suppression to former go and trained no-go cues
Source: Sci Rep. 2021 Jun 2;11:11544. doi: 10.1038/s41598-021-91040-6 (PMC8172546; doi:10.1038/s41598-021-91040-6)
Supplement: Supplementary file 1 — Supplementary Information. [file 41598_2021_91040_MOESM1_ESM.docx]

**Supplementary Materials**

**Experiment 1**

We additionally explored whether the effect of go and no-go colour assignment during Phase 1 had similar effects to the go and no-go cue assignment. To examine this, each of the six critical cues (2 × former go cues, 2 × trained no-go cues, and 2 × control cues) in Phase 2 were selected to be presented in the former go colour (e.g., blue) and trained no-go colour (e.g., yellow). Therefore, we had a fully crossed design with cue type and colour type in Phase 2 (see Supplementary Table 1) that allowed us to examine the effect the of colour assignment in Phase 1. To analyse the effect of colour, we calculated the mean MEP of all cues presented in the former go colour under low and high load separately; we did the same for all cues presented in the trained no-go colour. From these four conditions, we calculated a grand mean MEP value to normalise each condition for each participant (Figure S1).

Applying the same exclusion criteria for the cue type analyses there was one participant (three less) excluded for having normalised MEP outliers (> 3 standard deviations from the mean). There was no significant main effect of WM (F(1,54) = 0.16, p = 0.690, η^2^_p_ < 0.01), colour type (F(1,54) = 0.10, p = 0.749, η^2^_p_ < 0.01) or WM × colour type interaction (F(1,54) = 0.52, p = 0.472, η^2^_p_ = 0.01). There was no significant simple effect of WM on former go colour (t(54) = 0.74, p = 0.463, d = 0.10) or no-go colour (t(54) = 0.10, p = 0.921, d = 0.01). One sampled t-tests conducted on the log-normalised MEP comparing colour type at each WM level to a value of zero are presented in Supplementary Table 2.

The analyses revealed no strong effect of WM or colour type. One reason for not detecting an effect of colour type may be due to a prevalence for processing object information before colour information (e.g., Stroop, 1935). Alternatively, the method of normalisation to a grand average may not be sensitive enough to reveal differences in motor system excitability. Therefore, we decided not to pursue colour type further in Experiment 2 and all former go and trained no-go cues were presented in the same colour as Phase 1.

**Table S1.** Phase 2 design.

| **Cue type** | **Colour type** | **Trials in block (Experiment 1)** | **Trials in block (Experiment 2)** |
| --- | --- | --- | --- |
| Former go | Former go | 1 | 2 |
| Former go | Trained no-go | 1 | 0 |
| Trained no-go | Former go | 1 | 0 |
| Trained no-go | Trained no-go | 1 | 2 |
| Control | Former go | 1 | 1 |
| Control | Trained no-go | 1 | 1 |

**Figure S1.** Mean log-normalised MEP from Experiment 1 Phase 2 of a Go/No-go task for colour type (former go colour vs trained no-go colour) × working memory load (low vs high). Error bars represent within participant standard errors across working memory conditions separately for each cue type.

**Table S2.** Experiment 1 one sample t-tests (H_0_ = 0) of log-normalised MEP (Figure S1) for colour type x working memory load.

| **WM** | **Colour type** | **t** | **p** | **d** |
| --- | --- | --- | --- | --- |
| Low | Former Go | -0.874 | 0.386 | -0.118 |
|  | Trained No-go | -0.510 | 0.612 | -0.069 |
| High | Former Go | 0.398 | 0.692 | 0.054 |
|  | Trained No-go | -0.598 | 0.552 | -0.081 |
